# Intraluminal Farnesol and Farnesal in the Mealworm's Alimentary Canal: An Unusual Storage Site Uncovering Hidden Eukaryote Ca^2+^-Homeostasis-Dependent “Golgicrine” Activities

**DOI:** 10.3389/fendo.2019.00885

**Published:** 2019-12-19

**Authors:** Arnold De Loof, Liliane Schoofs

**Affiliations:** Functional Genomics and Proteomics Group, Department of Biology, KU Leuven, Leuven, Belgium

**Keywords:** juvenile hormone, insect hormones, Crohn's disease, inflammatory bowel disease, metamorphosis, mevalonate biosynthetic pathway, Vitamin D1, Alzheimer's disease

## Abstract

Farnesol, the sesquiterpenoid precursor of the six presently known insect juvenile hormones (JHs) was for the first time chemically identified in 1961, not in JH synthesizing glands or whole body extracts, but in excrements of the mealworm *Tenebrio molitor*. This finding was thought to be irrelevant and remained unexplored. In 1970, it was reported that the fall to zero of the JH titer in both prediapausing adults and in last instar larvae of the Colorado potato beetle causes severe malfunctioning of the Golgi system in the fat body, among various other effects. This endomembrane system in the cytoplasm resides at the intersection of the secretory, lysosomal, and endocytic pathways and is required for the processing of secretory proteins. Why the Golgi needs farnesol-like endogenous sesquiterpenoids (FLS) for its proper functioning has also never been further investigated. In 1999, farnesol was found to be a natural endogenous ligand for particular types of voltage-gated Ca^2+^ channels in mammalian cells, a finding that also remained undervalued. Only since 2014 more attention has been paid to the functional research of the “noble unknown” farnesol, in particular to its Ca^2+^-homeostasis-related juvenilizing and anti-apoptotic activities. Here, we introduce the term “Golgicrine activity” that addresses the secretory activity of the RER-Golgi system from its role in Ca^2+^-homeostasis rather than from its conventional role in mere protein secretion. Golgicrine activity attributes the so far forgotten role of farnesol-like sesquiterpenoids in proper Golgi functioning, and unites the endocrine, exocrine and enterocrine functions of these sesquiterpenoids. This out of the box view may open novel perspectives for the better understanding of particular inflammatory bowel diseases and of neurodegenerative diseases as well, because the early initiation of Alzheimer's disease may possibly result from malfunctioning of the mevalonate-farnesol-cholesterol biosynthetic pathway and thus might be a farnesol- and Ca^2+^-homeostasis-dependent Golgicrine issue.

## Introduction

For a long time, juvenile hormone (JH) of which to date six isoforms are known (1), was thought to be exclusively biosynthesized from farnesol in-, and secreted by the corpora allata (CA) of insects. This is the classical “endocrine JH”. When it was demonstrated that also the male accessory glands (MAGs) of the mosquitoes *Aedes aegypti, Anopheles rangeli, Anopheles trinkae*, and *Culex nigripalpus* (2) synthesize JH as does the ovary of *A. aegypti* (3), the CA lost their canonical exclusivity as JH production site. Twenty years later, Paroulek and Sláma (4) demonstrated that JH I present in the male accessory glands of the silk moth *Hyalophora cecropia* (5, 6), is not synthesized by the CA and subsequently transported to the MAGs, but instead synthesized by the MAGs themselves. The MAGs secrete it along with secretory proteins into the genital system of females during copulation. Paroulek and Sláma (4) functionally categorized this MAG-JH as “exocrine JH,” because it is not secreted into the hemolymph as a regular hormone. They speculated about its possible function(s) without, however, promoting a particular one. Perhaps, this “exocrine JH” is a first example of what we designate as “Golgicrine farnesol/FLS secretion”, a process that, in our opinion, likely occurs in all eukaryotes: see section Via Secretion of Digestive Enzymes in the Midgut Cells Through Their GOLGI Systems: “Golgicrine” Activity?

**Figure 1:**
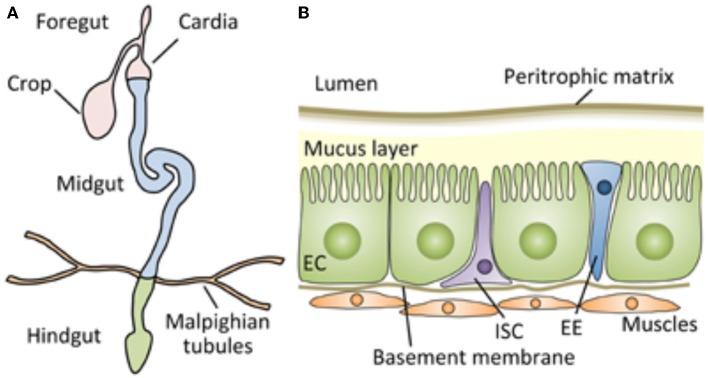
Schematic representation of the digestive tract of the *Drosophila* adult, which can serve as an approximate model for the architecture of the tract of most insects, including mealworms. **(A)** The *Drosophila* gut is a tubular epithelial organ composed of a monolayer of cells surrounded by muscles. The gut is divided into the foregut, the midgut, and the hindgut, based on their developmental origin. The midgut is the main site of digestion, and most studies of *Drosophila* gut immunity have focused on this compartment. **(B)** A cross section of the adult *Drosophila* midgut. From Kuraishi et al. (9). Open access under the terms of the creative Commons attribution 4.0 International license.

Few students and researchers are probably aware of the fact that the chemical identity of endocrine JH could only be elucidated thanks to pioneer studies by Schmialek (7), who worked with a most unusual model. Indeed, nearly 10 years before JH I was characterized in extracts of abdomens of male *Hyalophora cecropia* silk moths by Röller and Dahm (6), extracts of excrements (Kot in German) of the mealworm *Tenebrio molitor* were found to display prominent JH bioactivity when tested in JH-specific bioassays (8). In 1961, Peter Schmialek identified these active compounds in excrements as farnesol and farnesal. At that time, it was assumed that excrement farnesal and farnesol were degradation products of the still unidentified JH. The possibility that they might be functionally active inside the gut lumen was not considered. At the time, research on hormone receptors that explains the mode of hormonal action, was still in its infancy. Moreover, the site of synthesis of the farnesol and farnesal present in the gut (Figure 1) and excrements was also elusive.

The discovery that particular voltage gated Ca^2+^ channels act as membrane receptors for farnesol, as originally found in a mammalian system by Roullet et al. (10) and Luft et al. (11), can be marked as an important breakthrough. However, for a variety of reasons, its importance escaped the attention of most endocrinologists. Fifteen years later, in search for the causal mechanisms of programmed cell death, a process that occurs during complete metamorphosis of holometabolous insects, De Loof et al. (12, 13) introduced the concept of “the calcium-apoptosis link,” originally formulated by Orrenius et al. (14). That closed the circle: *absence of farnesol/FLS/JH* induces metamorphosis and massive programmed cell death (1). It does so via the mechanism of Ca^2+^-homeostasis in which Ca^2+^-induced Ca^2+^ release (from internal storage sites) plays an important role. Upon asking why not all tissues but only selected ones undergo programmed cell death during metamorphosis, the answer emerged that cells actively synthesizing and secreting proteins through their RER-Golgi system turned out to be the most “vulnerable” ones to die. Well-known examples of tissues undergoing programmed cell death and later developing anew from pre-existing stem cells are the midgut (but not the fore-and hindgut), the fat body (more or less the physiological equivalent of the vertebrate liver), the salivary glands and silk glands. De Loof (15) recently reported that the cells of such apoptosis-vulnerable tissues die because of the absence of endogenous farnesol-like sesquiterpenoids at the onset of metamorphosis. Apparently, *the main function of the RER-Golgi is to remove excess Ca*^2+^ *from the cytoplasm by secreting not just any type of protein, but by secreting Ca*^2+^*-binding/transporting proteins*. In addition, one of the targets of farnesol-like endogenous sesquiterpenoids is the Golgi system, with its special types of Ca^2+^-ATPases (16) and processing enzymes as was already shown in the Colorado potato beetle (17). The fact that thapsigargin, also a sesquiterpenoid, blocks the SERCA calcium pump suggests that this calcium pump has a binding site for endogenous farnesol-like sesquiterpenoids (12, 15, 18). In fact, the link between endogenous sesquiterpenoids and Ca^2+^ homeostasis represents an important change in paradigm in integrative cell biology (1).

With the cited novel insights from the past decades in mind, the results of Schmialek (7) deserve reconsideration and interpretation. Indeed, they may suggest an overlooked secretory role of the Golgi system for excreting endogenous farnesol-like sesquiterpenoids out of protein-secreting cells. The active secretion of calcium-binding proteins from the RER-Golgi might be accompanied by the secretion of farnesol or/and farnesol-like sesquiterpenoid substances (FLS) (see section Via Secretion of Digestive Enzymes in the Midgut Cells Through Their GOLGI Systems: “Golgicrine” Activity?).

The high farnesol/FLS concentration in the alimentary canal also suggests a role for maintaining gut cell integrity by its anti-apoptosis effect. It is possible that insects prevent the occurrence of inflammatory bowel diseases, not only by their short life or by a variety of immune defense strategies (9, 19, 20), but also by the creation of a high intraluminal farnesol/FLS concentration.

Given that the basic principles of cell biology, such as apoptosis, are almost universal in both vertebrates and invertebrates, this paper may open novel perspectives to gain a better insight in the possible causes of human bowel diseases such as Crohn's disease. Inflammatory bowel diseases are a global medical problem in the twenty-first century. The highest reported prevalence is registered in Europe and North America. Its incidence is rising in newly industrialized countries on all continents (21). The aim of this paper is to promote the idea that the serendipitous finding of Schmialek (7) might open novel avenues for studying possible causes and/or remedies for particular human diseases.

## Endocrine Archaeology: A Forgotten Discovery Done by Peter Schmialek in 1961

### Facts About the Discovery

**Figure 2:**
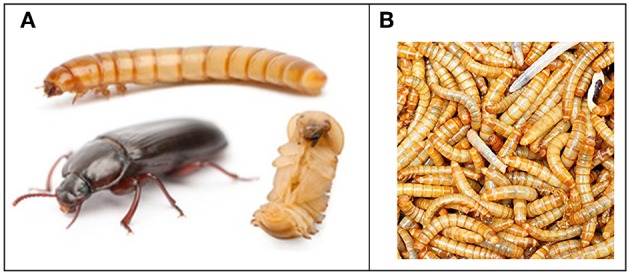
The mealworm *Tenebrio molitor*. **(A)** Life cycle showing larva, pupa and adult. Epic mealworms: Weebly page by students M. Downey, L. McFarland, and D. Bergeron, STU bachelor of Education 2014 (EDUC 5473). From Google images (no conditions for copyright published, apparently freely available). **(B)** Picture by Amazon of mealworm larvae as they are commercially marketed mainly for pet food. Open access.

Since the 1950s scientist have been looking for biological sources that display high activity when tested in specific bioassays for the detection of juvenile hormone. The aim was to find a source from which sufficient active material could be collected to engage in the chemical identification of JH. It was found that JH-active material was not only present in insects, but also in crustaceans and even in humans (22, 23). It soon became clear that the active substance might belong to the isoprenoids. It was Schmialek (7) who chemically identified the first JH-active substances, namely the isoprenoids farnesol and farnesal, in the common mealworm *Tenebrio* (Figure 2). These isoprenoid were not isolated and identified from whole body extracts nor from particular glands, but from extracts of *Tenebrio* excrements. The original paper was in German, at that time a common language in chemistry, physics, biology and engineering. The title “*Die Identifizierung zweier in Tenebriokot und in Hefe vorkommender Substanzen mit Juvenilhormonwirkung*” translated into English, reads: “The identification of two substances with Juvenile Hormone activity occurring in excrements of *Tenebrio* and in yeast.”

**Figure 3:**
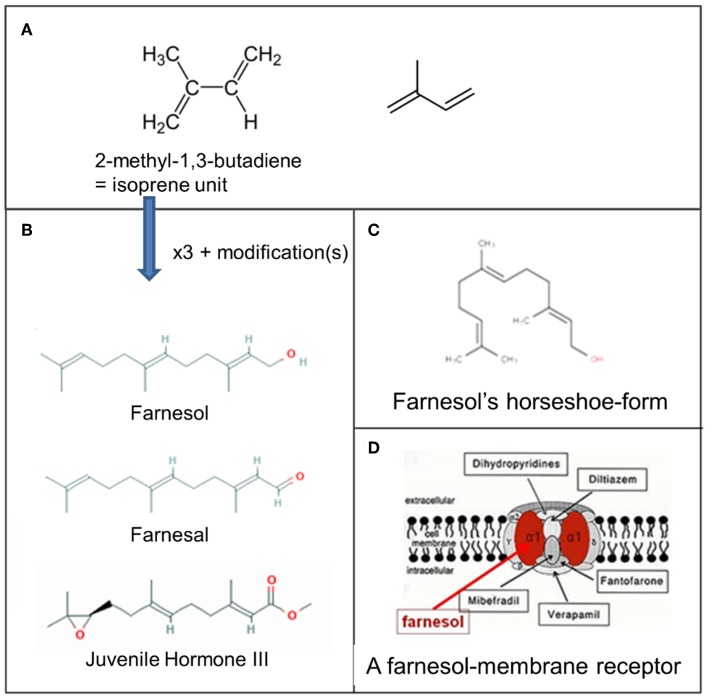
Farnesol: some chemistry. **(A)** Molecular structure of 2-methyl-1,3-butadiene, the isoprene unit from which sesquiterpenoids are biosynthesized (Wikipedia: Isoprene). Three such units are needed for the synthesis of farnesol, farnesal and the juvenile hormones. **(B)** Farnesol, Farnesal, and JH III, one of the esters of farnesol. **(C)** Horseshoe-shape of farnesol. **(D)** Schematic representation of one of the possible membrane receptors of farnesol, namely a voltage-gated Ca^2+^-channel in the plasma membrane with its binding site for farnesol in red. Copyright: **(A)** From Wikipedia: Sesquiterpene (Open Access). **(B)** From PubChem (Open Access); **(D)** Ca^2+^ channel (voltage gated)-adapted From Wikipedia: Calcium channel (adapted) and De Loof and Schoofs (1), Open Access.

The repeated extraction of 80 kg excrements of fully grown larvae of *Tenebrio* with methanol-benzol followed by absorption chromatography yielded 4 grams of a brown oily fraction with 40% (juvenile) hormone activity. Further purification by thin layer chromatography yielded 60 mg of a bright yellow oil substance that was 100% active in the same JH-bioassay, namely the *Tenebrio* Juvenile Hormone bioassay developed by Wigglesworth (24). The chemical identification proved that both farnesol and farnesal (Figure 3) were present in the fractions that displayed JH activity. For some reason, not mentioned in the paper, Schmialek (7) also extracted 12 kg of yeast. This extract, the weight of which was not reported, displayed 80% activity in the *Tenebrio* JH-bioassay. The identified active compounds, farnesal and farnesol, were identical to those identified in *Tenebrio* excrements. A likely reason for also extracting yeast will be dealt with in section From the Food? The Bacterial Flora?

Finally, Schmialek (7) observed that the *qualitative* effects of the identified farnesol and farnesal in the *Tenebrio* JH bioassay were similar to those provoked by enriched extracts of (abdomens of males of) the silk moth *Samia cynthia*. It took another 7 years before Juvenile Hormone I was chemically identified from an enriched extract of another silk moth, namely *Hyalophora cecropia* (6). Hence, due to the lack of synthetic JH I, it was impossible for Schmialek to compare their *quantitative* potencies in the JH bioassay. Neither was he able to discern whether farnesol and farnesal were true JHs, or merely degradation products of *Tenebrio*'s endogenous JH. Later, such comparative experiments were conducted by Wigglesworth (25). He observed that JH I was much more potent than farnesol. This turned out to be also the case for all other JH-isomers, which all turned out to be esters of farnesol.

### Chemical Properties of Farnesol and Mode of Action

Farnesol belongs to the family of the sesquiterpenes, a class of terpenes consisting of three isoprene units and in general represented by the molecular formula C_15_H_24_. Like monoterpenes, sesquiterpenes may be acyclic or contain rings, including several unique combinations. Biochemical modifications such as oxidation produce the related sesquiterpenoids. Sesquiterpenes occur naturally in plants and insects as semiochemicals, e.g., defensive agents or pheromones. Mass spectrometry has been used for their identification (26) and, within the methodological limitations, for quantification as well. De Araújo Delmondes et al. (27) recently summarized the toxicological and pharmacological effects of farnesol. In insects, farnesol is best known as a precursor of the six presently known JHs. The molecular weight (MW) of farnesol is 222.3 g/mol. Its rotatable bond count is 7, indicating that it is a very flexible molecule (1, 28). The best documented isomers of farnesol are: *trans, trans*-farnesol, *2-cis,6-trans*-farnesol, *2-trans,6-cis*-farnesol and *cis-cis*-farnesol (see PubChem), the all *trans* isomer being the most active in the *Tenebrio* JH-bioassay (25). The MW of JHs varies from 294.4 g/mol for JH I to 266.3 g/mol for JH III. Their respective rotatable bond counts are 10 for JH I vs. 6 for JH III. According to Wigglesworth (25), the effects scored by the various compounds in JH-bioassays *are not qualitatively but mainly quantitatively different*.

In vertebrate literature, it has been assumed for a long time that farnesol is neither a hormone, nor an “inbrome” (13). Instead, the general view was that farnesyl pyrophosphate only serves as a precursor for squalene in the mevalonate pathway, and that farnesol itself, even if it would occur freely in the cytoplasm or blood, has no particular function. That farnesol by itself can play a role in Ca^2+^ homeostasis has, however, been convincingly demonstrated by the electrophysiologists Roullet et al. (10) and Luft et al. (11). If farnesol by itself has a physiological function, the possibility exists that it fulfills the role of “juvenilizing agent” in vertebrates, including mammals and humans, much like farnesol and its JH-esters do in insects (13). As mentioned before, a problem for accepting this idea is that *in vertebrates* no bioassay is as yet available for investigating “juvenilizing activity” of compounds. Farnesol extracted from mammals nevertheless displays JH activity when tested in an insect bioassay for JH.

### Different Views on the Mode(s) of Action of Farnesol Esters With Juvenile Hormone Activity

To date, the most cited mode of action of JH (and farnesol as well) is that JH directs the whole physiology of its target cells through its binding to the transcription factor Germ cell-expressed (Gce) and its duplicate paralog Methoprene-tolerant (Met) in *Drosophila* (29). Although the advocates of this thesis do not deny the existence of a functional link between a possible membrane receptor and the activation of Met [e.g., (30, 31)], the role of a membrane receptor is often thought to be of minor importance, even to be non-existent. Considering the so far absence of convincing experimental evidence that JH can—and does—enter the nucleus, De Loof and Schoofs (1) recently proposed an alternative for the MET-mediated mode of action. Their counterarguments are that the Met model insufficiently takes into account that farnesol-like sesquiterpenoids are *hydrophobic* molecules that have a high affinity for lipidic membrane systems that are omnipresent in all target cells. This chemical property of farnesol and its esters has the following consequences. As soon as a farnesol/FLS molecule is secreted into the hemolymph, it associates with a lipoprotein by hydrophobic interaction. Next it is carried around in the body. When the JH-loaded lipoprotein comes in contact with the surface of any cell, some of the farnesol/FLS molecules will relocate into the lipid environment of the plasma membrane system. Because the lipid part of the plasma- and internal membranes is fluid, and because farnesol/FLS are small molecules compared to the size of the constituting lipids and proteins of the membrane, they will start diffusing over the entire connected membrane system of the cell. This way farnesol/FLS ends up in the RER, SER, Golgi, and nuclear envelope. Even the mitochondria are influenced (17). Thus, the *very first* key *acceptor* system of endogenous FLS is in fact *the membrane system*, and in particular, its lipid part. The explanation? For hydrophobic sesquiterpenoids and other hydrophobic molecules with a matching hydrophobicity profile, the lipid bilayer of biomembranes acts as a *solvent*.

This is only part of the story. Indeed, membranes harbor a large set of membrane proteins, many belonging to the family of helix bundle Trans Membrane (TM) proteins, with a variety of functions. Membrane proteins necessarily have stretches of hydrophobic amino acids, otherwise they could not reside in any membrane. Apparently, some of these membrane proteins have a binding pocket for farnesol, the first prerequisite for acting as a receptor for farnesol. Given the multitude of different (helix bundle) membrane proteins, small hydrophobic signaling molecules tend to be multifunctional. Many transmembrane proteins are involved in controlling the composition of the internal ionic environment of cells, of the ionic/electrical gradients over all membrane systems, of transport in or out the cell or subcellular compartments of macromolecules, GPCRs, enzymes with function in lipid-, steroid- and protein biosynthesis etc. Because some of these proteins have a binding site for farnesol/FLS, they are considered to act as membrane receptors for farnesol/FLS (12, 15). In contrast, how farnesol/JH end up in the watery environment of the nucleus is not yet known, but a lipidic transporter will anyhow be needed. In our opinion, the primordial receptor of not only JHs, but also of 20E, another key hormone in insect development, is *the Ca*^2+^*-homeostasis system in its entirety*.

### Methods of Application of Farnesol/FLS When Tested in Bioassays

The hydrophobic properties of farnesol and its esters impose certain recommendations as to how to apply them in bioassays. First, they have to be dissolved in organic solvents. They can be applied to the integument/skin from where they will diffuse into the body. Injection cannot be done in a watery suspension or along with wetting agents: such agents prevent positive reactions in bioassays (25). The recommended way is mixing farnesol/JHs with an oil. Inside the body, the oily mixture will act for a few days as a slow release formula (32). This indicates that there must be a continuous supply of farnesol/JH in the body for exerting biological activity.

## Possible Sources of Farnesol in Excrements

### From the Food? The Bacterial Flora?

Schmialek (7) explicitly stated that the excrements originated from fully grown (in German “erwachsene”) larvae. At the onset of metamorphosis, the larvae empty their gut. One possibility is that (a part of) the farnesol/farnesal originates from the food that mainly consists of plant material. Schmialek (7) also extracted yeast and found that it also contained farnesol and farnesal. We hypothesize that Schmialek performed this experiment as a control in order to exclude that the farnesol and farnesal he identified in the *Tenebrio* excrements would originate from the yeast present in the *Tenebrio* diet. Bits of yeast are indeed added to some diets for *Tenebrio* reared in laboratory conditions. The *Tenebrio* diet was not described in the Materials and Methods section of Schmialek's paper. *Tenebrio* survives perfectly with a yeast-free diet. Anyhow, we think that it is unlikely that all the JH-active material extracted from the excrements of *Tenebrio* originates from the ingested food. Indeed, farnesol is lipid-soluble. If it would be set free from the food during digestion, it would partition itself between the watery food phase and the lipid environment of the membranes of the gut cells. From there it would enter the hemolymph if its concentration would rise higher than the concentration of farnesol in the hemolymph. Such system would turn the alimentary canal into an endocrine gland that secretes farnesol into the blood, and in a way that fluctuates when the food composition changes. Such situation has never been documented in insects. Neither has it been shown that the microbiome in the insect gut might have a sufficiently active mevalonate biosynthetic pathway (which is absent in most prokaryotes) to account for the relative large amounts of farnesol in excrements as documented by Schmialek (7).

### Excreted via the Malpighian System?

Schmialek (7) favored the idea that farnesol and farnesal are degradation products of the (in 1961 still chemically unidentified) JH that circulates in the hemolymph of *Tenebrio* larvae. We also favor this hypothesis. However, one should not overlook the possibility that in addition to the farnesol ester JH, farnesol itself also circulates in the hemolymph of larvae. Indeed, Teal et al. (33) have shown that methyl farnesoate (MF) is a candidate hormone in at least five different orders of insects as it circulates in their hemolymph. It also does so in the swimming crab *Portunus trituberculatus* (34). According to Wen et al. (35), MF is produced by the larval CA and released into the hemolymph, from where it exerts its anti-metamorphic effects indirectly after conversion to JHB3, as well as by acting as a hormone itself through both nuclear JH receptors, Met, and Gce.

It is unusual in endocrinology that final degradation products of steroid- or peptide hormones still retain biological activity. A well-documented way of ecdysteroid degradation in insects is the formation of highly polar products, e.g., glucuronides, beta-glucosides, and sulfate conjugates with very reduced biological activity (36).

The usual system for excreting breakdown products as waste out of the body is their removal through the major excretory organ of most insect species, namely the Malpighian tubule system (Figure 1). It absorbs solutes, water, and wastes from the surrounding hemolymph. The wastes are released from the organism in the form of solid nitrogenous compounds and calcium oxalate. They are most likely not excreted via the Malpighian tubules. Which other option is left for explaining the presence of farnesol in the lumen of the midgut and in excrements?

### Via Secretion of Digestive Enzymes in the Midgut Cells Through Their Golgi Systems: “Golgicrine” Activity?

#### The History of the Term “Golgicrine Activity and Secretion” of Farnesol/JH

**Figure 4:**
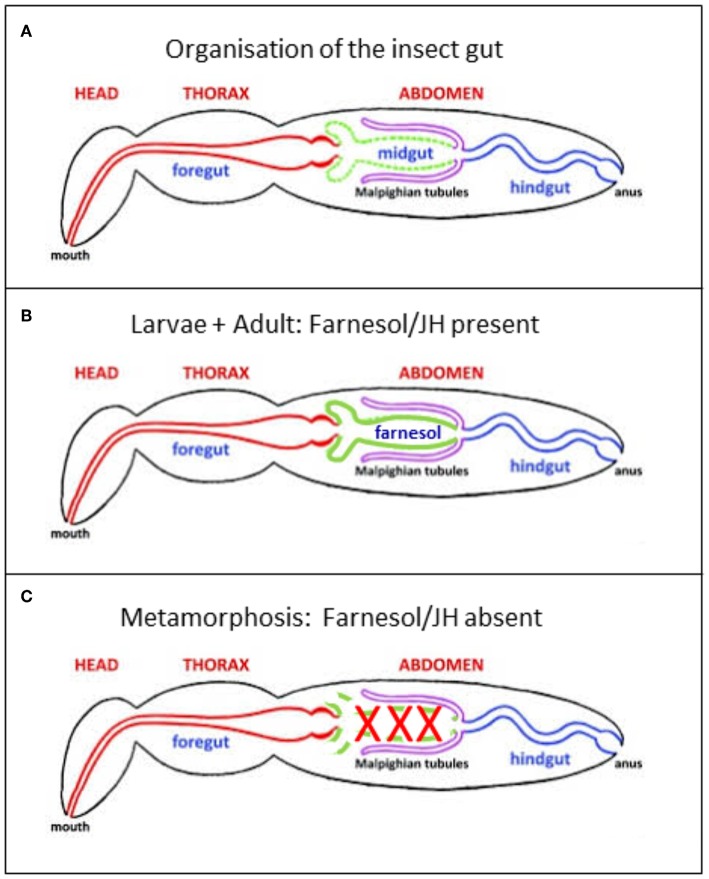
**(A)** Generalized insect digestive system illustrating the three main regions. Repeatedly modified from Imms (37). This version was modified by Michael L. Ferro. Posted on bugwood Wikimedia in 2011. **(B,C)** Further modifications. **(B)** In the presence of a high titer of endogenous farnesol-like sesquiterpenoids, in particular of JH, during young larval stages and during adult life, all parts of the alimentary canal remain intact. It has been shown by Schmialek (7) that farnesol and farnesal are present in the lumen of the gut of mealworm larvae. **(C)** When at the onset of the last larval instar the titer of endogenous farnesol-like sesquiterpenoids in the hemolymph drops to zero, the midgut starts undergoing programmed cell death/apoptosis. Next, it is remodeled into the adult gut, starting from stem cells.

Already 50 years ago, De Loof and Lagasse (17) published a paper on the changes in ultrastructure of fat body cells in adult females of the Colorado potato beetle (*Leptinotarsa decemlineata*) when the production of JH by the CA falls to zero under the influence of a short day photoperiod or by extirpation of the CA (more details in section The Colorado Potato Beetle as a Model to Visualize Cellular Functions in the Presence/Absence of Farnesol/FLS; Figure 4). The most obvious effect was altered secretory activity of the Golgi system resulting in the production and intracellular accumulation of large protein bodies. In the 1960–1970s, these effects were functionally interpreted as protein reserves for diapause and hibernation. How the *absence* of JH generated this effect was not investigated. That remained largely unchanged until Paroulek and Sláma (4) introduced the term “exocrine JH secretion” for denominating the secretion of JH biosynthesized in the male accessory glands (MAGs) of *Hyalophora cecropia*. Unlike “endocrine JH,” which is secreted by the CA into the hemolymph, the MAG's JH is not secreted into the hemolymph, but instead ends up, along with other secretory products of the MAGs, in the female during copulation. This finding shows that after its synthesis, JH is not distributed throughout the body by simple diffusion. Apparently, the MAG JH is transported by some mechanism of *polarized transport*. This interpretation of Schmialek's data also suggests polarized transport into the gut lumen. The only possibility we see for effectuating such transport is through the RER-Golgi system.

One possible scenario for explaining the presence of farnesol in excrements may read as follows. During larval life, the JH-active materials, farnesol and JHs, secreted by the CA, enter the integrated (plasma- and connected intracellular) membrane systems of the gut cells, like they enter all cell membranes. The most important function of the RER-Golgi system, which is a target of JH (17), is the secretion of (Ca^2+^ binding/transporting) proteins packed in membrane-coated vesicles. As such, the RER-Golgi of midgut cells produces vesicles that contain digestive enzymes. Another scenario could be that the midgut cells produce farnesol/FLS themselves, and that by means of hydrophobic interactions these sesquiterpenoids have a stronger affinity for both the cargo proteins present in the RER-Golgi and the membranes of the secretory vesicles than for the lipoproteins present in the hemolymph.

In both scenarios, this means that willy-nilly the fluid membrane coating of these vesicles contains farnesol/FLS molecules that freely float around in membrane systems. It is widely assumed that at the moment of secretion, the limiting membrane of the vesicle fuses with the plasma membrane, and that only the vesicle content will be discharged into the gut lumen. If the fusion process is 100 percent effective, part of the farnesol/FLS present in the membrane translocates into the interior of the vesicle where it attaches to the digestive enzymes-containing content. This may result from hydrophobic interactions. Thus, farnesol is willy-nilly secreted along with the release of vesicles from the midgut cells.

#### Secretion of JH by the CA: Also a Role for the RER-Golgi System?

If the description in section The History of the Term “Golgicrine Activity and Secretion” of Farnesol/JH is correct, it means that not only the Golgi system of midgut cells but that of all types of cells with an active protein secretion Golgi machinery do the same. The *corpus allatum* is not known as an actively protein secreting gland, but according to Bradley and Edwards (38), the typical CA cells of the house cricket *Acheta domesticus* possess large nucleoli, active Golgi complexes, numerous mitochondria, and occasional microtubules. In the earwig *Euborellia annulipes lucas*, Golgi bodies, and endoplasmic reticulum are poorly developed (39). The exact role of the RER-Golgi system in JH production is not known. Perhaps, it produces (cargo) proteins packed in vesicles that bring the newly synthesized JH to the plasma membrane ready for secretion into the hemolymph. One should not a priori reject the possibility that the Golgi system is a site of synthesis of “exocrine” farnesol/JH. This implies that farnesol/JH is subj*ect to unidirectional (polarized) export out of the cell*. Thus, contrary to what has been assumed up to the present day, it may not leave the cell by simple random diffusion through the plasma membrane of the CA cells into the hemolymph. Perhaps, *unidirectional secretory transport* of farnesol/FLS is a common feature in Golgi systems of some, if not all eukaryotes. If it is, this concept may become a game changer in several ways. Indeed, *it redirects the activity of the Golgi from a key role in the secretion of (cargo) proteins out of the cell, into a key role in the removal of excess Ca*^2+^ *out of the cytoplasm, and this with the help of Ca*^2+^*-binding/transporting proteins and of farnesol/FLS*. In addition, as long as the identity of the protein(s) secreted through the RER-Golgi is not known, the suggestion by Sláma (40) that this secretion product might have an important physiological function is a valid one. One of Sláma's arguments was that not a sesquiterpenoid, but a synthetic *peptide* he tested in a bioassay, was by far the most active compound with JH bioactivity. In our opinion, farnesol/FLS may be ubiquitous and abundant hormones in all animals, humans inclusive. If true and confirmed by future experiments, this would be the opposite of the way they are perceived to date, namely intermediates in the mevalonate biosynthetic pathway.

#### Endogenous Farnesol-Like Sesquiterpenoids: A Role in Maintaining Membrane Integrity?

Active Golgi systems are characterized by continuous membrane remodeling. This implies that the membranes must be very flexible and instantly self-closing upon contact with fusing vesicles or when vesicles are pinched off. The plasma membrane should therefore be very flexible but nonetheless tough. Perhaps endogenous sesquiterpenoids facilitate these processes.

## A Function(s) for Farnesol Inside the Gut's Lumen?

### Gut/Excrement-Farnesol Is Still Biologically Active in Assays for Juvenile Hormone: an Intriguing Observation

Intuitively one might think that farnesol ending up in excrements is the final result of the clearing of such compounds from the body. It is unusual in endocrinology that final degradation products of steroid- or peptide hormone still retain biological activity. A well-documented example in insect endocrinology is the way of ecdysteroid degradation by the formation of highly polar products, e.g., glucuronides, beta-glucosides, and sulfate conjugates (36). Hence, it is intriguing that farnesol in excrements is equally biologically active in bioassays for JH as farnesol from any other source. Apparently “gut farnesol” is not an “end of the line” breakdown result: further breakdown to full inactivity is possible, but that does not happen for all farnesol present in the midgut's lumen. In our opinion, the persistence of the biological activity of farnesol and farnesal in the gut lumen suggests a function. Which one?

### The Insect Midgut of Holometabolous Insects Completely Degenerates During Metamorphosis: Apoptosis

As long as the JH titer in young larvae and adults of holometabolous insects is high, the whole alimentary canal with its three major parts, fore-, mid-, and hindgut (Figure 4) functions well: no programmed cell death. Fore- and hindgut are of ectodermal origin. Only the midgut is of endodermal origin.

When the JH titer drops to zero at the onset of the last larval instar, the functionality of the midgut gets jeopardized, no matter what the diet of the species was (herbivore, carnivore, dead wood, wool etc.). Food ingestion stops and the midgut is induced to undergo programmed cell death/apoptosis. In insects with an incomplete metamorphosis, the JH titer never drops to zero, and all parts of the gut remain intact all the time. Correlation or causal? During metamorphosis of holometabolous insects, the midgut is completely remodeled starting from stem cells after the larval cells have died. In many species, the adult alimentary canal can handle a diet that is very different from the larval one. For example, many caterpillars, which are larvae from Lepidoptera, are herbivorous in larval life, whereas the adults, butterflies or moths, are often feeding on nectar.

### Does, Perhaps, Farnesol on Its Way to Be Excreted Have an “Enterocrine Anti-apoptotic” Function?

A physiological function for farnesol/FLS inside the alimentary canal was not considered in Schmialek's paper, probably because at that time insect endocrinologists had other priorities, in particular the identification of the chemical identity of “insect juvenile hormone.” Midgut cells are vulnerable to damage, and to an untimely influx of excessive Ca^2+^. To date and to our knowledge, nobody as yet reported any function or beneficial or deleterious toxic effects of farnesol/FLS in any part of the alimentary canal in any insect- or non-insect species. One of the reasons is that, although present in all eukaryotes, farnesol hitherto remained a “*noble unknown*” to many cell biologists and endocrinologists. The main reason for this poor documentation on a nonetheless important molecule, which is produced in the mevalonate biosynthetic pathway that is operational in all eukaryotes, is that *in vertebrates farnesol is neither known as a hormone, nor as an “inbrome”* (1, 13), nor as a neurotransmitter, nor as any other type of signaling molecule. No specific vertebrate-type bioassay for farnesol is known, this in sharp contrast with the situation in insects where farnesol, and even more its juvenile hormone esters, are known to be master hormone systems in controlling development (see next). It is neither well-known that farnesol plays a role in GPCR signaling through its role in prenylation of G-proteins (28). Prenylation is important, e.g., for RAS protein functioning from yeast to humans (41).

Considering the fact that farnesol is a potent blocker of Ca^2+^ channels as was reported by Roullet et al. (10) and Luft et al. (11), and that high titers of farnesol/JHs/FLS inhibit apoptosis (1), intriguing questions emerge. The key one is: Whatever the source of farnesol and farnesal in the gut lumen is, could farnesol-like endogenous or exogenous (from food e.g.) sesquiterpenoids have a protecting function against programmed cell death of the insect midgut cells (42) that invariably occurs in all species undergoing complete metamorphosis?

A first aspect of the answer is that the purification of farnesol from the excrement extracts was monitored by a bioassay that detected juvenile hormone activity in *Tenebrio* itself. Thus, a homologous bioassay was used, thereby excluding false positive results that would possibly occur using a heterologous assay. Second, an anti-apoptotic effect was experimentally documented by Dai and Gilbert (43), who observed that the degeneration of the prothoracic glands of *Manduca sexta* was inhibited following injection of JH.

From the wording in his paper, it can be inferred that Schmialek (7) was aware of the existence of farnesol-isomers with differential biological activity. Which isomer(s) Schmialek (partially?) purified was not mentioned, probably because the methods for determining the stereoisomeric identity were not available. In 1969, Wigglesworth reported that the all-trans isomer (*trans, trans*-farnesol; (E,E)-farnesol: terminology in PubChem) is the most active one. It is exactly this isomeric form that is the most active in JH bioassays (25). Roullet et al. (10) also found that it is the all-trans-farnesol isomer that is present in the human brain and that it is this isomeric form that acts as a blocker of N-type voltage-gated Ca^2+^ channels in mammalian models.

## An Anti-apoptotic Function for Farnesol/FLs: Omnipresent In Eukaryotes, Humans Inclusive?

Given that farnesol/FLS and Golgi activity are omnipresent in eukaryotes, it is improbable that the midgut story during in insect metamorphosis is a unique feature. Rather, it might be a common phenomenon, in particular in cells with a very active RER-Golgi.

### Absence of Farnesol/JH Particularly Targets Cell Types With an Active RER-Golgi System: Consequences

Since Schmialek's (7) paper, our knowledge on programmed cell death and apoptosis in general cell physiology has enormously increased. In particular the concept of Orrenius et al. (14), namely “Regulation of cell death: The “Calcium-apoptosis link” incited De Loof (15) to search for the physiological explanation why only selected tissues undergo apoptosis/programmed cell death, while others continue to further develop. The main tissues undergoing programmed cell death are: the midgut (but not the fore- and hindgut), the salivary glands, and the fat body (which functions as both the liver and adipose tissue of vertebrates). Which property do they have in common? The midgut is of endodermal origin, the others are of mesodermal origin. Thus, the developmental origin is not causal to be sensitive to programmed cell death. Their functions as an organ are very different: no clue either. It finally became clear that those tissues that are very sensitive to the drop of zero of farnesol/FLS all have in common that they actively secrete proteins via their RER-Golgi systems. Having in mind the Orrenius et al. (14) concept, the question emerged how this could be related to Ca^2+^ homeostasis. A publication by Vandecaetsbeek et al. (16) reporting that the Golgi system has a special set of Ca^2+^-ATPases did De Loof (15) consider the possibility that, perhaps, the main function of the RER-Golgi system is *not* the secretion of any type of (cargo) protein of which the functionality is always outside the cell, somewhere else in (or even out) the body, but *the removal of toxic excess cytoplasmic Ca*^2+^ *by means of Ca*^2+^*-binding/transporting cargo proteins*. This view drastically changes the picture: it highlights excess Ca^2+^ as the true inducer of apoptosis.

**Figure 5:**
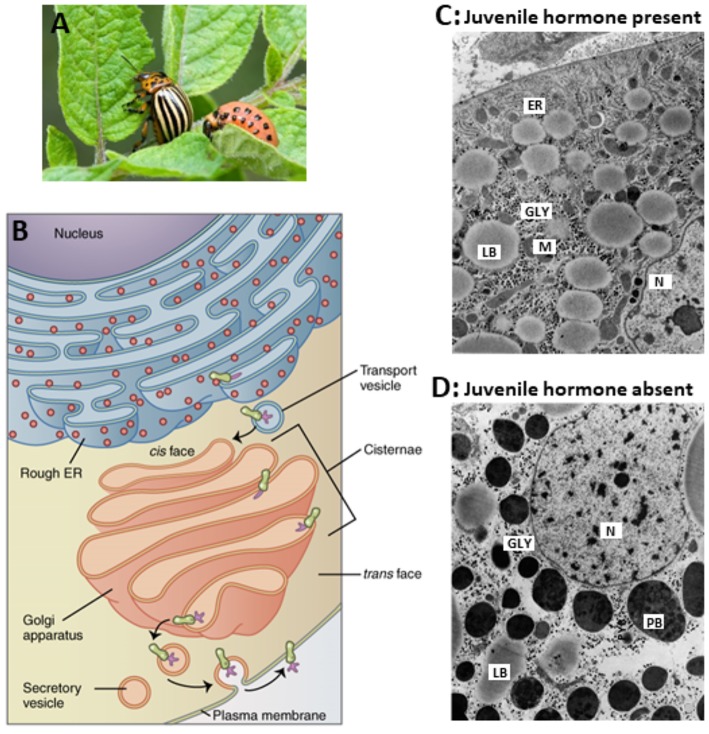
Comparison of the ultrastructure of fat body cells of two adult females of the Colorado potato beetle, that were forced to undergo hypertrophy of the whole fat body under two different regimes. **(A)** Larva and adult. **(B)** RER-Golgi system. **(C)** This beetle was reared under long-day conditions which activate the *corpora allata* to produce JH. In addition, the beetle was ovariectomized. Thus, the hypertrophy happened in the presence of JH. **(D)** This beetle was reared under short-day conditions which inactivate the *corpora allata*. Thus, here the hypertrophy happened in the absence of Juvenile hormone. The comparison shows that in the absence of JH, there is an enormous accumulation of protein bodies (PB) containing “storage or diapause proteins” which result from the fact that the normal processing of secretory vesicles in the Golgi apparatus **(B)** gets jeopardized. How the Golgi is a target for JH is not known. In the absence of JH, very few mitochondria are present. ER, rough Endoplasmic Reticulum; GLY, glycogen; M, mitochondrion; N, nucleus; LB, lipid body; PB, protein body. Copyright: **(A)** from Google images, Open Access. **(B)** From Wikipedia: Golgi: Open Access. **(C,D)** Slightly modified from figures 70 and 71 in “Aggregaatsthesis” of De Loof (44) (Open Access). Additional information, e.g., on lysosomal activity, can be found in De Loof (44, 45) and in De Loof and Lagasse (17).

That the absence of JH has a drastic effect on the secretory activity and capacity of the Golgi system had been demonstrated by De Loof and Lagasse (17) many years ago in the Colorado potato beetle, as is illustrated in Figure 5. The major issue is that in the absence of endogenous sesquiterpenoids, some proteins that are normally secreted out of the cell, cannot leave the cell anymore, but instead accumulate in the cytoplasm as larger storage vesicles. In Figure 5 they are named “protein bodies.”

If the interpretation of De Loof (15) that the main function of the RER-Golgi system is to remove toxic excess Ca^2+^ from the cytoplasm, the question may be asked whether a malfunctioning farnesol-Golgi system might, perhaps, be causal to various poorly understood human diseases. In this paper we limit ourselves to briefly discussing why we think that this malfunctioning system might, perhaps, be instrumental to the triggering of induction of Alzheimer's disease. Because we are not medics but biologists specialized in animal physiology and endocrinology, we only focus on cell physiological aspects of the disease. This topic is discussed at length elsewhere (46).

For researchers, who have little or no experience with comparative research insects vs. mammals (humans inclusive), advancing a model that originated from physiological research in insects dating from nearly half a century ago that tries to uncover a major cause of induction of Alzheimer's disease may be too far sought. Indeed, the gross differences are substantial. The average lifespan of humans is about 80+ years. Depending on the species and the environmental conditions, insects live for only a few weeks or months. Insects do not develop Alzheimer's disease-like symptoms when aging as adults. The two canonical markers of Alzheimer's disease, amyloid-beta plaques, and tau tangles (47) do not start occurring in aging insects. Insects undergoing complete metamorphosis do enter a coma-like phase from which they wake up just before adult eclosion. The main similarity resides in the occurrence of massive apoptosis/cell death. This process occurs in various tissues in insects while in humans, apart from the cells of the adaptive immune system and the continuous renewal of gut cells, it is mainly restricted to the brain of Alzheimer's disease patients. However, the principles of apoptosis are the same in invertebrates and vertebrates. This made us ask the question whether a dropping farnesol production might somehow be causal to the induction of Alzheimer's disease. The first question to be answered concerns the occurrence of farnesol in the human brain. If absent, the hypothesis that apoptosis in the degenerating brain cells might be a result of a declining farnesol/FLS concentrations is invalid. The second one concerns the question which cellular functions are controlled by farnesol in vertebrates/mammals?

### Is the Mevalonate Pathway Active in the Human Brain and/or in Other Tissues?

Elaborating on this hypothesis only makes sense if farnesol/FLS occurs in human brain cells. Few data are already available and indicate that this indeed the case. Roullet et al. (10) reported that farnesol is naturally occurring in brain tissue of humans and rodents. The stereoisomer they identified in both human and rodent brain was the *all-trans*-farnesol isomer. *Cis, trans*-farnesol was not detectable. Farnesol concentrations were estimated to range from 373 to 417 pmol/g wet weight. The measured values in young rat brain were around 590 pmol/g (wet weight). In human necropsy specimens (frontal cortex) from 4 individuals (46 years old or older) who died from different causes and all analyzed <24 h. Post-mortem, *all-trans*-farnesol was detected in all samples in the range of 110–290 pmol/g (fresh weight). In rats, farnesol is present in the blood and prostate gland as has been reported in a preliminary study by De Loof et al. (13).

### Which Cell Physiological Functions Are Controlled by the “Noble Unknown” Farnesol in Vertebrates/Mammals/Humans?

Only few publications on farnesol in a medical-physiological context in humans can be retrieved from PubMed (1). The reasons have been listed before (section Does, Perhaps, Farnesol on Its Way to be Excreted Have an “Enterocrine Anti-apoptotic” Function). One of the major reasons is that in vertebrates many cell types can engage in activities of the mevalonate pathway. Thus, there is no single cell type or organ that has exclusivity for producing farnesol and its derivatives. The quantitative output may, however, differ among cell types. The second one is that vertebrates do not have a developmental stage in which the mevalonate pathway becomes totally inactivated, thus allowing observation of the changes that occur when farnesol/FLS is completely cleared from the body. This contrasts sharply with the situation in insects, in particular in those that undergo complete metamorphosis. Indeed, in *larval* insects, farnesol/FLS biosynthesis is predominantly restricted to a pair of tiny glands, named the *corpora allata*. These glands can be surgically removed (= known as allatectomy) from the body. This results in a fast drop in the titer of farnesol/FLS, and even in total absence of farnesol/FLS. Because such treatment is not lethal in the short term, one can observe which cellular functions change as the result of the transition from “farnesol/FLS present” to “totally absent.” In addition to allatectomy, there is also a natural physiological process resulting in total inactivity of the CA, namely metamorphosis. This drastic process is initiated when the endogenous production of farnesol/FLS is endocrinologically blocked. Several mechanisms bring about such block. A similar arrest of farnesol/FLS biosynthesis also occurs in adults of some species preparing for entering diapause (hibernation), a stage in which the animals become totally inactive, e.g., under the influence of changes in daylength. A good example is the Colorado potato beetle, as will be dealt with next.

### The Colorado Potato Beetle as a Model to Visualize Cellular Functions in the Presence/Absence of Farnesol/FLS

#### Subcellular Changes in Fat Body Cells Caused by the de Declining JH Titer in the Colorado Potato Beetle

In the Northern hemisphere the daylength starts decreasing from midsummer on. This change in photoperiod stops reproduction in adults, and it induces the physiological preparations for entering *diapause*, a state of inactivity that lasts at least one winter. One of such changes is the *hypertrophy of the fat body*. After about two weeks after the trigger has been activated, the animals stop eating, burrow into the soil, and they become immobile. Because of a low metabolic rate they can survive in this dormant condition until at least the next summer. An essential element of the survival is the accumulation of large amounts of reserves: lipids, glycogen, and a special type of proteins, named “diapause proteins,” which are synthesized in the RER and secreted in abnormal high amounts by the Golgi system. Some of these diapause proteins remain in the fat body cells, others end up in the hemolymph, and accumulate there in high concentrations. Short daylength conditions somehow inactivate the *corpora allata* (CA). As a result, the titer of JH drops to zero. Thus, in this condition the subcellular changes preceding apoptosis of fat body cells during diapause are caused by the declining JH titer. That this CA inactivation is causal to the described cellular changes is proven by the fact that surgical removal of the CA yields the same effects as the ones induced by short day conditions. Furthermore, application of synthetic JH mixed in oil temporarily rescued the effects of allatectomy and of short day conditions.

In long day conditions, the CA keep on actively producing and secreting JH. But there is a second way to make the fat body hypertrophy in adults, namely, namely ovariectomy. This allows the electron microscopic comparison of the conditions “JH present” vs. “JH completely absent” as shown in Figure 5 (44). The most conspicuous difference is the appearance of large proteid bodies (PBs) in the absence of JH. Apparently, absence of JH jeopardizes the normal functioning of the Golgi system. In the presence of JH, the Golgi secretes proteins present in small secretory vesicles. In long day conditions, the secretory vesicles of reproducing females mainly contain yolk precursors (vitellogenin and lipoprotein). In short day conditions, vitellogenin production is halted. Now, “diapause proteins” are synthesized. The comparison of both conditions shows that absence of JH causes changes in the multiplication of mitochondria, in lipid- and glycogen metabolism, in jeopardized “Golgicrine” activity, in changes in nuclear and cytoplasmic architecture. Other changes, some not observable by electron microscopy are changes in the types of proteins that are secreted, in particular the diapause proteins (45), other in the increased production of lysosomal enzymes (44), in behavior, in metabolism, and in ecdysteroid metabolism (48).

When analyzing the changes that occur in the fat body of pre-metamorphosing last instar larvae of the Colorado beetle, thus in larvae with inactive CA, De Loof (45) observed that these changes were the same as those he observed in short day-reared adults. This resulted in the formulation of “Diapause phenomena in non-diapausing last instar larvae. This made us hypothesize that, perhaps in evolutionary retrospect, the AD situation may be a partial remnant of an ubiquitous mechanism to enter and survive diapause/hibernation, but that unlike insects humans miss a good cellular renewal system involving stem cells.

It can be expected that a lowered production and secretion of farnesol/FLS in the human brain may likewise yield negative multifunctionality. The jeopardized Golgicrine activity and the massive induction of apoptotic activity in the brain make us suggest that investigating the role of the mevalonate pathway in Alzheimer's disease may yield novel insights, and hopefully, better prevention and treatment.

Insects do not undergo massive apoptosis in their brain, but they do in other tissues, in particular in situations where the farnesol/JH titer drops to zero (metamorphosis and in insects with JH-controlled diapause). In our opinion, in humans, there is a situation, namely Alzheimer's disease, in which a similar system as shown in Figure 5 might be at work (46).

#### Insects Manage to Survive Situations of Massive Programmed Cell Death/Apoptosis: How?

From a medical viewpoint it looks like a miracle that insects in which the midgut, the fat body and some other tissues nearly completely degenerate, nonetheless survive and continue to develop in another form, the adult, which is able to reproduce as if nothing had happened. In its totality, this is unimaginable in mammals. Yet, for some tissues, regeneration is “daily practice,” e.g., the intestinal epithelium is the fastest renewing tissue in mammals, humans inclusive (49). Insects survive because they manage to clear the body of all the end products of the lysed tissues, while stimulating regeneration of new tissues starting from preexisting dormant stem cells. Exactly this ability to sufficiently regenerate from quiescent stem cells is missing in the Alzheimer's patients brains, although good progress is being made (50–53). The stem cells may need growth factors, but their identity remains largely unclear. Yet, an interesting novel insight has recently been advanced by Sláma (54). He suggested that the molting hormone of insects and other arthropods, namely 20-hydroxyecdysone the titer of which is high during metamorphosis, is a water-soluble anabolic steroid. Remarkably, although vertebrates do not synthesize this steroid, it yields the same anabolic effects in insects, quails and in humans (55–57). Sláma (54) renamed 20E as Vitamin D_1_. Vitamin D_1_, which occurs in large concentrations in some plants (*Leuzea* e.g.) and which is commercially available, is legally used in geriatrics and by sportsmen to increase their muscular mass.

#### Are Results Obtained in Insects Extrapolable to Mammals, Humans Inclusive?

There is no straight yes-no answer to this question. It depends upon how essential a given process is. Some cell physiological principles have been shaped to near perfection in the course of evolution. For example the principles of the “central dogma,” some minor differences not taken into account, are very well-conserved since billions of years. A similar situation is present with respect to the Ca^2+^ homeostasis system. In some of its possible forms the mevalonate biosynthetic pathway was already present in the choanoflagellates, which are considered ancestral to all animal species (1). The fundamental principles of programmed cell death are also very well-conserved, also biochemically. Thus, we think that fundamental physiological processes occurring in insects are extrapolable to vertebrates and *vice versa*. Ca^2+^-induced programmed cell death (14) and “Ca^2+^-induced Ca^2+^ release” (58, 59) are probably two of them. Of course, experimental validation of the supposed similarities remains necessary. At least, the comparison insects-vertebrates may yield novel ideas as to how to uncover the still largely hidden roles of farnesol/FLS in mammals/humans. In this respect, *Drosophila* has been used as a model in research on Alzheimer's disease (60–62). In a forthcoming paper, our arguments in favor of a putative role of a dysfunctional farnesol-producing mevalonate biosynthetic pathway as a master-inducer of Ca^2+^ dependent deleterious changes in brain cell physiology of Alzheimer's patients are outlined in De Loof and Schoofs (46).

## The Situation in Alzheimer's Sick Brain Cells: Partially Similar to Insect Cells that Enter the Cell Death Pathway During Metamorphosis?

### Amyloid and Tau Protein Malfunctioning: The Cause or Rather the Result of Alzheimer's Disease?

**Figure 6:**
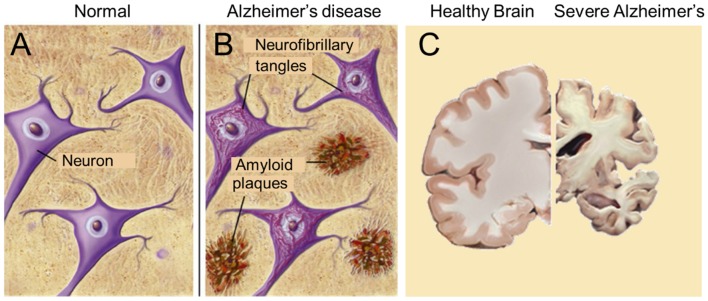
The major pathological hallmarks of the disease are amyloid plaques and neurofibrillary tangles **(B)**, which are absent in healthy brain tissue **(A)**. Massive apoptosis occurs in later stages of Alzheimer's disease in the human brain (brain slices) **(C)**. Sources: **(A,B)** from Google Images, BrightFocus Foundation (Alzheimer's disease: Amyloid Plaques and Neurofibrillary tangles). **(C)** Wikimedia, from Elsevier Sci. Tech connect. The neural basis of Alzheimer disease. Posted by Caitlin Beddows, 2016. All figures are Open Access.

According to De-Paula et al. (63), Alzheimer's disease (AD) is a chronic neurodegenerative disease with well-defined pathophysiological mechanisms, mostly affecting the medial temporal lobe and associative neocortical structures. Neuritic plaques and neurofibrillary tangles (Figure 6) represent the pathological hallmarks of AD, and are, respectively, related to the accumulation of the amyloid-beta peptide (Aβ) in brain tissues, and to cytoskeletal changes that arise from the hyperphosphorylation of microtubule-associated Tau protein in neurons. See also “The Alzheimer's disease Fact sheet” from NIH/NHA and video fragments available on the internet. For over two decades, research on AD has largely concentrated on protein malfunctioning, in particular of the cited amyloid-beta and Tau. One of the possible ways in which Aβ becomes toxic is by incorporating into neuronal membranes forming calcium-permeable ion channels resulting in abnormal elevation of intracellular calcium levels (64). Aβ and tau-directed research, amidst several other approaches, was conducted worldwide in numerous laboratories and commercial companies, but it has now come to an almost complete standstill. Since at least a decade researchers have started to doubt whether the “protein line of search for causal agents” was the right avenue. In other words, are the “canonical supposed causal proteins amyloid and tau” rather the *result* than the cause of the disease [culprit or coincidence: (65)?] But, if the “result” hypothesis would hold, which then is the real primary cause of the disease? Nobody can at present answer this question. Several possibilities have been advanced over the years, e.g., a prominent role for Ca^2+^-dyshomeostasis and neurotoxicity (64, 66, 67), but both remain subject for debate.

Innovative fresh ideas about overlooked possibilities might be welcomed. From our experience with insect physiology, in particular with respect to the mechanisms operating during metamorphosis in which apoptosis of specific cells/tissues plays a major role, we think that researchers working on AD may gain profit from learning how insects overcame, many millions of years ago, a major apoptosis problem due to a jeopardized mevalonate-farnesol biosynthetic pathway. *The trick: keep the titer of farnesol/FLS high, and apoptosis will not be induced*.

### The Role of the Golgi System in a Neuron

**Figure 7:**
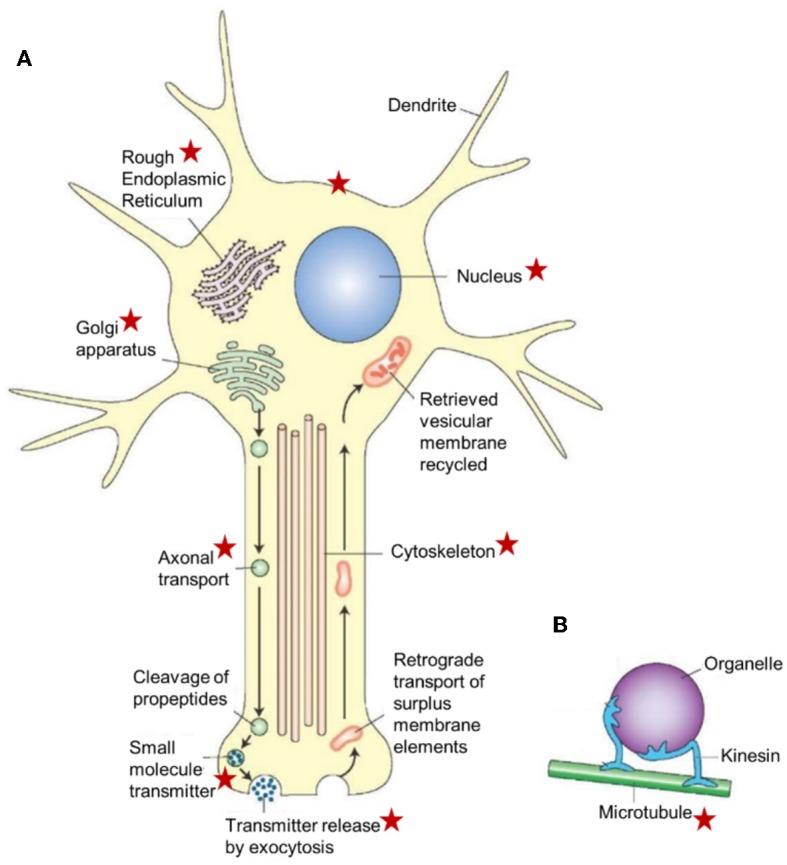
Schematic representation of a neuron illustrating the major cellular sites and cell organelles that are influenced by endogenous farnesol-like sesquiterpenoids, as highlighted by the red asterisks. This list is not exhaustive (e.g., the figure does not list the mitochondria which are known targets of farnesol/JH). The functional relation between farnesol/FLS and the Ca^2+^-homeostasis system with its numerous actors, explains why there are so many target sites. This figure is combinatorial: the asterisks represent data from different experimental models. The basic idea underlying this figure is also valid for other cell types, in particular for those with an active RER-Golgi system. This figure, borrowed from Google pictures, specifically focuses on axonal transport. **(A)** Large peptide molecules (pre-propeptides) are converted into smaller peptides (propeptides) in the rough endoplasmic reticulum. The propeptides and enzymes are packaged into vesicles that are transported to the Golgi apparatus, modified, and packaged into vesicles. The vesicles get attached to microtubules and are carried to the terminals by fast axonal transport. The propeptides are cleaved into smaller peptidergic transmitters in the axon's terminal. The peptide neurotransmitters are released into the synaptic cleft by exocytosis. Surplus membrane elements in the terminal are carried back to the cell body by retrograde transport. The retrieved vesicular membrane is degraded or recycled. **(B)** A model showing how kinesin (a microtubule-associated ATPase) can move an organelle along a microtubule. Copyright information: This figure is listed in Google Images, without specific information on the authors, only general information. From: What-when-how; In Depth Tutorials and Information; Histology of the Nervous System (The Neuron) Part 1, The Neuron figure 5-2. With thanks to the anonymous author(s). For lack of information, Open Access is assumed.

The main function is the production and packing of neurotransmitters (of all sorts) in vesicles that are transported inside the neuron with the help of the cytoskeleton (Figure 7). In the presynaptic part of the synapse, the vesicles fuse with the plasma membrane and release their contents into the synaptic cleft. In disease conditions, Tau mislocalizes into pre- and post-synaptic compartments (61, 62). Mattson et al. (68) reported that the endoplasmic reticulum appears to be a focal point for alterations that result in neuronal dysfunction and death in AD, but a possible role for the involvement of the mevalonate pathway was not mentioned. If it is a universal effect that a drastic decrease of the titer of farnesol/FLS jeopardizes the secretory activity of the Golgi (17). As illustrated in Figure 5, synaptic functioning is likely to become impaired. This may be one of the deleterious effects of a malfunctioning mevalonate pathway. A second major effect is the induction of apoptosis. A third one is malfunctioning of the cytoskeleton, because it depends on a well-functioning Ca^2+^-homeostasis system like many other cellular functions, including all steps in protein synthesis. Thus, because the neuron contains the subcellular structures and the signaling pathways that are sensitive to lower concentrations and even total absence of farnesol/FLS, the negative multifunctionality of a dysfunctional mevalonate pathway may be causal to some of the AD symptoms.

**A few suggestions for future research, in particular in vertebrates**

1. Quantify the farnesol/FLS titer in blood in order to determine if farnesol/FLS may act as a hormone.
2. Find out if all cells/tissues with an actively secreting Golgi-system release farnesol/FLS? If that would be the case, it would be a true game changer in cell physiology. Indeed, it would promote farnesol/FLS to the most ubiquitous hormone/signaling molecule of the animal kingdom.
3. Determine which stereoisomers are present in the human body.
4. Develop a vertebrate bioassay(s) for farnesol/FLS, e.g., based upon its antagonist or agonist function on some elements of the Ca^2+^-homeostasis system.
5. Measure farnesol/FLS concentrations in normal and Alzheimer's brains. Which isomers are present?
6. Is the mevalonate pathway under control of some neuropeptide(s) like it is in insects?
7. Feed farnesol/FLS to model-mammalian models or apply them to the skin, and determine the parameters of their uptake into the body.
8. Uncover the mode of action of farnesol/FLS at the level of the Golgi system. Do the “special” Ca^2+^-ATPases present in the Golgi (16) play a role? What about a functional relationship between farnesol/FLS and secretases? Is, perhaps, “exocrine” farnesol/FLS/JH that so far has only been described in the reproductive system of both males and females of a few insect species [refs in De Loof and Schoofs (1)], a common feature of cells/tissues that actively secrete (cargo) proteins?
9. Investigate whether Vitamin D_1_ (54) might act antagonistically or synergistically to endogenous farnesol-like sesquiterpenoids.

## Discussion

Gut cells are vulnerable to damage, and to an untimely influx of excessive Ca^2+^. Has in addition to the “endocrine” and “exocrine” activities of farnesol/FLS (4), its putative “enterocrine” function been overlooked? Or is the latter just a variant of the “exocrine system”? Can the “*Golgicrine* farnesol/FLS” concept be put forward as the overarching umbrella of all three, exocrine, endocrine and enterocrine activities? Is it possible that endogenous farnesol-like sesquiterpenoids play an overlooked role in innate insect immunity, in particular in the gut's immune system? Next, because farnesol is omnipresent in all eukaryotes, as are Ca^2+^-channels, might it also have function in some parts of the healthy vertebrate/human alimentary canal? If so, this may open novel perspectives for improved treatments of (inflammatory) bowel diseases, such as Crohn's disease in humans. In addition to the cited emerging questions, this paper also tries to answer the still only partially answered question on how endogenous sesquiterpenoids act at the molecular level. Is it only at the level of transcription (29, 31) and/or on the whole integrated system of Ca^2+^-homeostasis (1, 28, 69)?

From a cell-physiological “economical” point of view, using farnesol/farnesal inside the alimentary canal as an anti-apoptotic agent would be a clever strategy. Indeed, as long as the titer of farnesol-like compounds with juvenile hormone activity is high in the hemolymph, the juvenile life phase is continued, the gut remains intact, and aging processes are suppressed and delayed. When farnesol/farnesal titers drop to zero, as happens at the onset of metamorphosis, apoptosis results.

It is indeed striking that one of the compounds found in excrements of *Tenebrio* (7) is identical to farnesol, the sesquiterpenoid that has both juvenile hormone activity and antigating activity on Ca^2+^ channels (10, 11). Farnesal was not tested on Ca^2+^-channel activity by Roullet et al. (10). Thus, given that the concentration of farnesol is high enough, a physiological (protective?) role seems to be a possibility.

From Schmialek's paper, the origin of farnesol in excrements cannot be deduced. Food is one of the theoretical possibilities. All plants contain farnesol-like sesquiterpenoids (7). If their concentrations in plants would suffice to protect the insect midgut against apoptosis, the same might also hold true for herbivorous vertebrates, humans inclusive. Since we now know that the mevalonate biosynthetic pathway producing farnesol operates in all eukaryotes, any diet will contain farnesol-like endogenous sesquiterpenoids. Depending on the diet, only the farnesol/FLS concentrations will vary. It is possible that anti-apoptosis activity only occurs above a critical threshold concentration of farnesol/FLS. Maybe, the natural concentrations of farnesol in any diet of mammals, herbivorous or carnivorous, is too low to prevent or cure some types of bowel diseases. Addition of exogenous *trans, trans*-farnesol deserves being tested. Added at 20 g/kg diet, farnesol is not toxic when orally ingested as reported in a study on the (beneficial) effect of treating a patient with pancreas cancer (70). Remarkably, the limited number of follow-up publications (listed in PubMed) on this topic suggests that oncologists, for one reason or another and rightly or wrongly, do not see great potential for large scale practical applications of farnesol in cancer prevention (71) and treatment.

While the causal link between the absence of farnesol/JH and a malfunctioning Golgi system was already documented in insects in 1970 by De Loof and Lagasse, we are currently unaware of any publication on this issue in any vertebrate system. The probable reason is that in comparative physiology and endocrinology in the 1960–1970s, comparative invertebrate (insect)—vertebrate (mammals) research was not readily welcomed. Insects were thought to be very primitive and simple compared to the supposedly highly advanced mammals. Now we known that the opposite holds true: due to their short life cycle and high rate of reproduction, insects are millions of generations ahead of mammals in evolution. They specialized in miniaturization, while mammals embraced the idea that “big is beautiful.” That they would share the same evolutionary ancient basic principles of cell physiology, and that these were very well-conserved in the various progenies of the common ancestor, only gradually infiltrated the field of comparative physiology.

For non-specialists in insect endocrinology, it is worth mentioning that about 4,000 analogs of farnesol/JHs have been synthesized in the past decades, not in a medical context, but in aiming at developing novel insect growth regulators that might be used as insecticides with very low toxicity for most vertebrates (40, 72). Many of these compounds, developed in this search for novel insecticides, act as growth regulators, and are much more active in JH-bioassays, up to a million times, than farnesol. Their toxicity to vertebrates is mostly low. A few have been commercialized. Van Mellaert et al. (73) reported on the anti-JH effect of some synthetic benzoylphenols that caused sterility in female insects (inhibition of ovarian development) without being mutagenic like other types of chemosterilants are. In signaling systems in which farnesol acts through prenylation (28), just adding farnesol to the diet may not suffice in conditions where the enzymes needed for prenylation are absent. *All-trans* farnesol (also named farnesol EE) is commercially available in large quantities at reasonable prices. Recently, the anti-Alzheimer's disease activity of four novel sesquiterpenoids using the *C. elegans* AD pathological model have been reported (74).

Farnesol/JHs are known to act antagonistically toward 20-OH-ecysone (46), the insect molting hormone. During metamorphosis the titer of ecdysteroids rises to high concentrations and contributes to the remodeling of the body, while the titer of JHs falls to zero. Recently, Sláma et al. (54) has put forward the concept “vitamin D_1_” for presenting the *water-soluble anabolic steroid* of plant origin (55) that also displays “molting hormone” activity in insects and crustaceans. This real vitamin D occurs in high concentrations in some plants. It is effective in both mammals, humans, and insects. Since a few decades it is legally used in medicine (in particular in geriatrics) and in sports. It is readily commercially available and it is relatively cheap. The ongoing debate in literature regarding the effect of vitamin D3 in patients with inflammatory bowel disease (75–79) may benefit from this recent insight in vitamin D research.

Making a jump from “farnesol in excrements of a beetle” to formulating a hypothesis “on the early origin of Alzheimer's disease in humans” may look too far sought, at first encounter at least. Insects are not known to suffer from an Alzheimer-disease like syndrome in their brain. However, like Alzheimer's disease patients suffer from massive apoptosis in their brain, metamorphosing insects similarly suffer from apoptosis in several tissues. Induction of apoptosis has a causal link with dysregulation of Ca^2+^-homeostasis. Insects survive tissue degeneration with the help of their mevalonate biosynthetic pathway, in particular by using farnesol and some of its esters for keeping intracellular Ca^2+^ levels low. Humans do produce farnesol, but hitherto its functions are “noble unknowns” in vertebrates in general, humans inclusive. In our opinion, exploring the *multifunctionality* of the mevalonate biosynthetic pathway in the context of neurodegenerative diseases [e.g. Alzheimer's or Parkinson's diseases (68, 80, 81)] may yield promising perspectives, in particular in ways to restore calcium homeostasis (66). Some of the neurodegenerative changes, which in the past were ascribed to mutations, may be the “simple” result of a suboptimal functioning mevalonate pathway.

In conclusion: Schmialek (7) did not elucidate the chemical nature of “insect juvenile hormone,” but he paved the way for further experiments demonstrating that all known JHs are esters of farnesol. Our paper is meant to be a late tribute to him for his scientific achievements that almost 60 years later still stimulate the development of novel physiological concepts.

## Author Contributions

AD and LS jointly conceived and wrote the paper.

### Conflict of Interest

The authors declare that the research was conducted in the absence of any commercial or financial relationships that could be construed as a potential conflict of interest.
